# Effects of physiological and manipulative loading conditions on the sacroiliac joint: a three-dimensional finite element analysis

**DOI:** 10.3389/fbioe.2026.1906528

**Published:** 2026-10-19

**Authors:** Juntao Ma, Xuanyu Zhou, Shuyan Xu, Languo Dai, Yuting Wang, Junlong Lin, Dingkun Lin, Ji Qi

**Affiliations:** 1 The Second Clinical College of Guangzhou University of Chinese Medicine, Guangzhou, China; 2 Chinese Medicine Guangdong Laboratory, Zhuhai, China; 3 State Key Laboratory of Dampness Syndrome of Chinese Medicine, Guangzhou, China; 4 Department of Orthopedics, Guangdong Provincial Hospital of Chinese Medicine, Guangzhou, China; 5 Orthopedic Biomechanics Laboratory, Guangdong Provincial Hospital of Chinese Medicine, Guangzhou, China

**Keywords:** biomechanics, finite element analysis, iliolumbar ligament, manipulative loading, sacroiliac joint

## Abstract

Manual therapy is applied in the conservative management of sacroiliac joint (SIJ)-related symptoms, but whether its biomechanical effect is primarily related to joint mobilization or periarticular soft-tissue modulation remains unclear. This study compared the mechanical responses of the SIJ-iliolumbar ligament complex under four prescribed loading and constraint configurations: physiological compressive stress (PCS), traction manipulation stress (TMS), oblique-pulling manipulation (OPM), and counter-directional sacral thrust with iliac mobilization (CDST-IM) using three-dimensional finite element analysis. A CT-based lumbar spine-pelvis finite element model was reconstructed from computed tomography data of a healthy 31-year-old male volunteer. Four loading conditions were simulated: 300 N downward compression at L4, 300 N vertical traction at L4, 300 N traction combined with 10° posterior rotation of the right ilium, and 600 N posterior force applied to the right ilium along the SIJ direction. von Mises stress (VMS), Total Deformation (TD), and range of motion (ROM) of the SIJ, together with iliolumbar ligament VMS and TD, were evaluated. PCS and TMS showed little difference in the evaluated responses under bilateral iliac constraint. OPM generated the greatest cartilage VMS, cartilage TD, right SIJ ROM, and iliolumbar ligament TD, whereas CDST-IM produced a more moderate SIJ response with comparatively low ligament TD. On the right side, the surface showing greater cartilage TD changed from the sacral surface under PCS/TMS to the iliac surface under OPM/CDST-IM. These findings indicate limited SIJ micromotion and distinct ligament responses under the prescribed loading and constraint configurations.

## Introduction

Low back pain is a major cause of disability worldwide, and the sacroiliac joint (SIJ) is increasingly recognized as a clinically important pain generator in a substantial proportion of affected patients. Epidemiological and diagnostic reviews have reported that SIJ-related pain accounts for approximately 15%–30% of low back pain cases, and the disorder is frequently overlooked because its clinical presentation overlaps with discogenic, facet-mediated, and other lumbopelvic pain syndromes (Buchanan et al., 2021; Newman and Soto, 2022; Kiapour et al., 2020).

Because SIJ dysfunction is commonly managed conservatively, non-operative care remains the first-line strategy and usually includes patient education, exercise-based rehabilitation, pelvic stabilization, and manipulative therapy (Newman and Soto, 2022; Trager et al., 2024; Nejati et al., 2019; Javadov et al., 2021). Among manual techniques used for SIJ dysfunction (Zhang et al., 2018; Zhu et al., 2012), oblique-pulling manipulation (OPM) has been used in clinical practice. During OPM, the patient is positioned in side-lying, with the dependent leg extended and the upper leg slightly flexed. The practitioner stands at the patient’s ventral side, stabilizes the posterior sacrum with one hand and contacts the anterior superior iliac spine on the treated side with the other. After positioning, the practitioner maintains sacral stabilization while applying an oblique traction–rotation action that guides the treated ilium posteriorly (Xu et al., 2020; Li et al., 2022; Xu et al., 2023b). Counter-directional sacral thrust with iliac mobilization (CDST-IM) has been described for a dysfunction pattern characterized by posterior sacral rotation and anterior iliac rotation. The patient is positioned in side-lying, with the dependent hip and knee flexed and the upper leg extended posteriorly. The practitioner stands behind the patient, places the heel of one hand over the middle of the sacrum, and controls the upper anterior superior iliac spine with the other hand. After relaxation of the lumbopelvic region, the two hands act simultaneously as a counter-directed force couple, pressing and stabilizing the sacrum anteriorly while drawing the treated ilium posteriorly (Long et al., 2004; Long and Fan, 2019).

However, the biomechanical mechanism of these manipulations remains controversial. One view proposes that SIJ manipulation restores lumbopelvic stability by directly mobilizing or repositioning the SIJ, whereas another suggests that the clinical benefit is mediated primarily through periarticular soft tissues rather than gross articular correction (Xu et al., 2020; Pool-Goudzwaard et al., 2003; Tullberg et al., 1998). This controversy is anatomically and biomechanically plausible because the SIJ is a highly constrained joint with very limited physiological motion, and prior stereophotogrammetric analysis showed that manipulation did not measurably alter the position of the sacrum relative to the ilium (Kiapour et al., 2020; Tullberg et al., 1998). At the same time, cadaveric research has demonstrated that the iliolumbar ligament contributes importantly to SIJ stability, and sequential transection of this ligament significantly increases sagittal SIJ mobility (Pool-Goudzwaard et al., 2003).

Previous biomechanical studies have begun to clarify the effects of SIJ manipulation, but most have focused on single manipulations or isolated loading scenarios rather than direct comparison of multiple loading strategies. Existing experimental and computational evidence indicates that SIJ biomechanics are highly sensitive to both loading mode and ligament integrity (Xu et al., 2020; Li et al., 2022; Lindsey et al., 2014; Eichenseer et al., 2011). Our previous finite element studies further suggested that OPM and CDST-IM may produce distinct mechanical response patterns in the SIJ-iliolumbar ligament complex (Zhou et al., 2025; Shi et al., 2026). However, these studies assessed each manipulation separately and did not fully characterize interface-level cartilage displacement across loading conditions.

Therefore, the present study was designed to compare physiological compressive stress (PCS), traction manipulation stress (TMS), OPM, and CDST-IM, with particular emphasis on SIJ articular cartilage and the iliolumbar ligament. The objective was to compare SIJ motion, cartilage stress, sacral and iliac cartilage displacement, and iliolumbar ligament responses across four prescribed loading and constraint configurations.

## Materials and methods

### Data collection and ethics

A 31-year-old healthy male volunteer weighing 60 kg underwent a computed tomography (CT) scan at our hospital. Imaging confirmed no osseous abnormalities-no pelvic fractures, deformities, or tumors. A Siemens dual-source CT scanner (SOMATOM Definition) was used for scanning, with a tube voltage of 120 kV, a tube current of 220 mA, a slice thickness of 5 mm and an interslice spacing of 5 mm. The scan extended from the superior margin of the L4 vertebra to the middle of the femur, thus covering the entire ischial tuberosity. Subsequently, the images were resampled as 1.0-mm slice thickness and exported in DICOM format.

This study was approved by the Ethics Committee of Guangdong Provincial Hospital of Chinese Medicine (approval no. BM2022-059) and was conducted in accordance with ethical standards for human research.

### Three-dimensional finite element model development

The DICOM data were imported into Mimics Research 21.0 for image segmentation. Bony structures were extracted using threshold-based segmentation, and cortical bone boundaries were then manually refined. The polygonal surface model was further imported into Geomagic 21.0 for surface smoothing and defect repair. After optimization, the model was converted into solid geometry in STP format using SolidWorks 2021, where the intervertebral discs, articular cartilage, and ligamentous structures were assembled. Finally, preprocessing and meshing were performed in ANSYS 2021R1. The solid structures were meshed using 10-node quadratic tetrahedral elements (SOLID187). The finite element model consisted of 2,032,220 nodes and 1,269,454 elements (Figure 1). At least three elements were assigned through the thickness of the SIJ cartilage, with local mesh control and gradual mesh transitions applied around the cartilage and ligament-bone attachment regions. The iliolumbar ligament bundles were reconstructed according to anatomical landmarks, extending from the lower lumbar transverse processes to their attachment regions along the iliac crest. The SIJ cartilage was constructed geometrically in SolidWorks.

**FIGURE 1:**
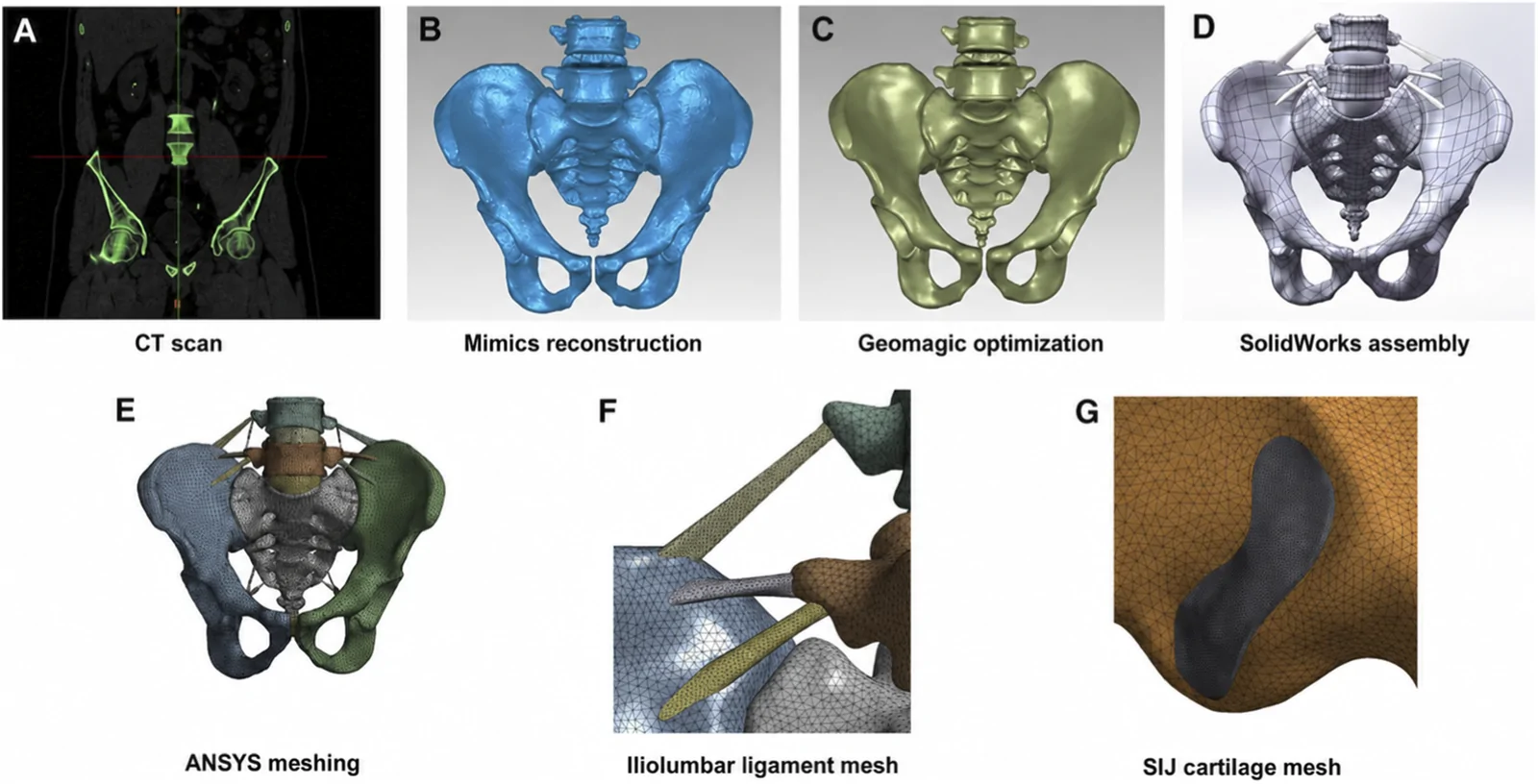
Workflow for construction of the three-dimensional lumbar spine-pelvis finite element model. **(A)** CT scan; **(B)** Mimics reconstruction; **(C)** Geomagic optimization; **(D)** SolidWorks assembly; **(E)** ANSYS meshing; **(F)** iliolumbar ligament mesh; **(G)** SIJ cartilage mesh.

### Material properties

Material properties were assigned on the basis of previously published biomechanical and finite element studies (Table 1). The iliolumbar ligament was modeled as six irregular three-dimensional solid bundles following anatomy, with lengths of 36.50 mm (left upper), 20.06 mm (left middle), 25.74 mm (left lower), 38.11 mm (right upper), 17.65 mm (right middle), and 23.33 mm (right lower). For simplification, the anterior sacroiliac, posterior sacroiliac, interosseous sacroiliac, sacrotuberous, and sacrospinous ligaments were modeled using tension-only spring elements, which transmitted tensile but not compressive forces. Articular cartilage was constructed within the joint space to reproduce the cartilage morphology, with a thickness of approximately 2 mm. The surface-to-surface contact at the SIJ cartilage-sacrum interface was defined as no separation to maintain contact during loading: normal separation was prevented after contact was established, and frictionless tangential motion was allowed (friction coefficient = 0) (Zhou et al., 2025; Shi et al., 2026).

**TABLE 1:** Material properties used in the finite element model and their literature sources.

Structure	Model type	Elastic modulus (MPa)	Poisson’s ratio	Stiffness (N/mm)	References
Cortical bone	Solid	12,000.00	0.30	N/A	Bruna-Rosso et al. (2016); Xu et al. (2020)
Cancellous bone	Solid	100.00	0.20	N/A	Bruna-Rosso et al. (2016); Xu et al. (2020)
Intervertebral disc endplate	Solid	24.00	0.40	N/A	Shahraki et al. (2015)
Annulus fibrosus	Solid	4.20	0.45	N/A	Shahraki et al. (2015)
Nucleus pulposus	Solid	1.00	0.49	N/A	Shahraki et al. (2015)
Pubic symphysis	Solid	5.00	0.45	N/A	Xu et al. (2020)
Spinal cartilage	Solid	10.00	0.40	N/A	Lindsey et al. (2015)
Sacroiliac joint cartilage	Solid	100.00	0.30	N/A	Xu et al. (2020)
Iliolumbar ligament	Solid	75.00	0.30	N/A	Guo and Li (2020)
Anterior longitudinal ligament	Spring	N/A	N/A	8.74	He et al. (2024)
Posterior longitudinal ligament	Spring	N/A	N/A	5.83	He et al. (2024)
Ligamentum flavum	Spring	N/A	N/A	15.38	He et al. (2024)
Supraspinous ligament	Spring	N/A	N/A	2.39	He et al. (2024)
Interspinous ligament	Spring	N/A	N/A	10.85	He et al. (2024)
Intertransverse ligament	Spring	N/A	N/A	0.19	He et al. (2024)
Articular capsular ligament	Spring	N/A	N/A	15.75	He et al. (2024)
Posterior sacroiliac short ligament	Spring	N/A	N/A	400	Hu et al. (2017)
Posterior sacroiliac long ligament	Spring	N/A	N/A	1,000	Hu et al. (2017)
Arcuate pubic ligament	Spring	N/A	N/A	500	Hu et al. (2017)
Superior pubic ligament	Spring	N/A	N/A	500	Hu et al. (2017)
Anterior sacroiliac ligament	Spring	N/A	N/A	700	Hu et al. (2017)
Sacrospinous ligament	Spring	N/A	N/A	1,400	Hu et al. (2017)
Sacrotuberous ligament	Spring	N/A	N/A	1,500	Hu et al. (2017)
Interosseous sacroiliac ligament	Spring	N/A	N/A	2,800	Hu et al. (2017)

Solid indicates structures modeled using solid elements; spring indicates ligaments represented using tension-only spring elements; N/A indicates not applicable.

### Kinematic comparison and local cartilage mesh assessment

The kinematic response of the model was evaluated by comparison with previously published biomechanical data reported by Lindsey et al. (2014) and Zhang et al. (2022). To simulate the single-leg stance condition, the left ilium was fixed and a 7.5 N·m moment was applied to the superior endplate of L4 to reproduce flexion-extension, lateral bending, and axial rotation. The range of motion (ROM) of the sacroiliac joint (SIJ) under these loading conditions was then compared with the published results to assess agreement with the published data.

Peak cartilage VMS sensitivity was assessed under the OPM condition using nominal local cartilage mesh sizes of 1.5, 1.0, and 0.5 mm. The corresponding models contained 1,777,142, 2,032,220, and 2,200,075 nodes and 1,101,693, 1,269,454, and 1,384,412 elements, respectively. The 1.0-mm local cartilage mesh corresponded to the model used for the reported analyses. ROM and TD were not separately assessed for convergence, and no whole-model mesh-convergence analysis was performed.

### Boundary conditions and loading conditions

A static structural analysis with Large Deflection enabled was used to characterize the equilibrium response at each prescribed terminal loading state.

1. PCS loading: Both iliac sides of the lumbar spine-pelvis three-dimensional finite element model were fully constrained to prevent relative displacement during the analysis. A 300 N downward force was applied to the superior endplate of L4 along the direction of body weight to simulate the mechanical effect of the upper body mass on the SIJ under physiological loading (Figure 2A).
2. TMS loading: Both iliac sides of the lumbar spine-pelvis three-dimensional finite element model were fully constrained to prevent relative displacement during the finite element analysis. A 300 N upward force was applied to the superior endplate of L4 along the direction opposite to body weight to simulate the mechanical effect of traction on the SIJ (Figure 2B).
3. OPM loading: The lateral side of the left ilium and the posterior side of the sacrum were fully constrained to simulate fixation of the lateral position during manipulation and to prevent sliding of the left ilium during the finite element analysis. A 300 N upward force was applied to the superior endplate of L4 along the direction opposite to body weight, and a prescribed 10° posterior rotation was applied to the right ilium through a remote displacement boundary condition. This terminal angle was selected based on our previous cadaveric OPM study, in which 300 N traction was combined with 10° posterior rotation (Li et al., 2022) (Figure 2C).
4. CDST-IM loading: The lateral side of the left ilium and the posterior side of the sacrum were fully constrained to represent the fixed state during manipulation and to prevent displacement of the left ilium during the finite element analysis. A 600 N posterior force was applied to the surface of the right ilium along the SIJ direction to simulate the relative motion between the sacrum and the right ilium during CDST-IM (Shi et al., 2026). The 600 N load was selected based on previous measurements of SIJ manipulation forces, in which forces recorded from five therapists averaged approximately 600 N (Xu et al., 2020; Xu et al., 2023a) (Figure 2D).

**FIGURE 2:**
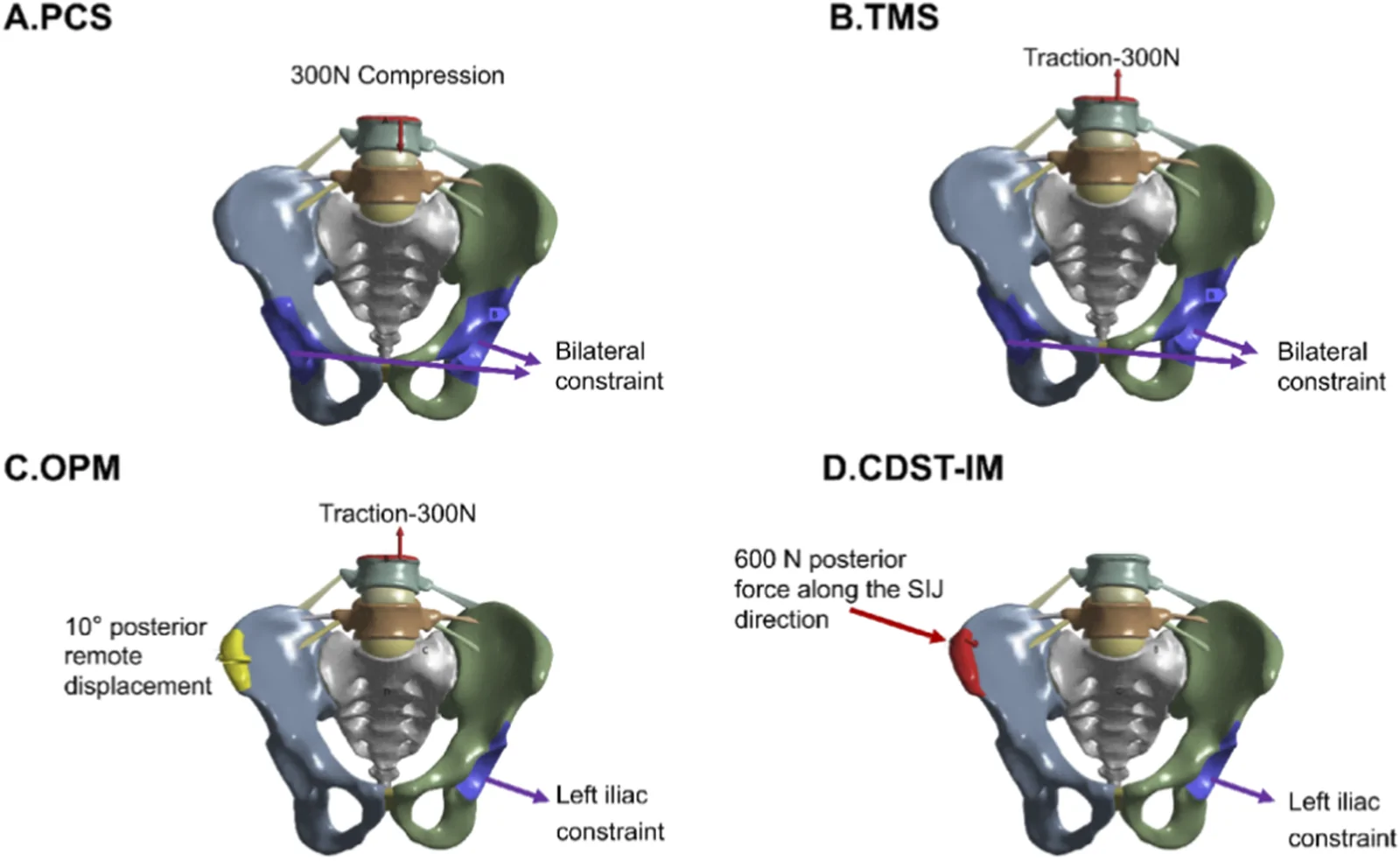
Boundary conditions and loading configurations for the four simulated conditions. **(A)** PCS: 300 N downward compression was applied at L4 with bilateral iliac constraint. **(B)** TMS: 300 N upward traction was applied at L4 with bilateral iliac constraint. **(C)** OPM: 300 N upward traction was applied at L4 combined with 10° posterior rotation of the right ilium, with the left ilium and sacrum constrained. **(D)** CDST-IM: 600 N posterior force was applied to the right ilium along the SIJ direction, with the left ilium and sacrum constrained. Red arrows indicate loading direction, and purple arrows indicate constrained regions.

### Data analysis

For each loading condition, von Mises stress (VMS) and Total Deformation (TD) were extracted from the SIJ articular cartilage and the iliolumbar ligament. Peak values were recorded for overall comparison. VMS, TD, and ROM were selected to compare multiaxial stress magnitude, displacement magnitude, and relative joint rotation, respectively, under the same model and material definitions. TD represents the magnitude of nodal displacement relative to the initial configuration. TD was analyzed separately for the iliac-side and sacral-side cartilage surfaces on both the left and right sides. The reported SIJ ROM was obtained from the change in angular displacement extracted at a remote point placed in the SIJ cartilage region between the initial and terminal loading states. For the iliolumbar ligament, TD was evaluated both globally and separately for the upper, middle and lower bundles on both sides. Maximum and minimum principal stresses were additionally extracted from the SIJ cartilage to characterize its tensile and compressive stress components. For the iliolumbar ligament, the maximum principal stress field was evaluated, and its maximum and lowest displayed values were recorded to characterize the distribution of tensile and compressive stress states. In addition, maximum directional displacement was extracted along the assigned local X-axis corresponding to the longitudinal direction of each ligament bundle. This loading-direction-specific measure was used to characterize bundle-level motion associated with the tensile response of the iliolumbar ligament.

## Results

### Kinematic comparison and local cartilage mesh results

The lumbar spine-pelvis finite element model was evaluated by comparing the calculated SIJ ROM under flexion-extension, lateral bending, and axial rotation with previously published cadaveric and finite element data from Lindsey et al. (2014) and Zhang et al. (2022). The calculated ROM values were generally within or close to the reported ranges, with only mild deviations in left SIJ flexion-extension and axial rotation. These results showed reasonable agreement with the published SIJ kinematic data and provided a kinematic reference for subsequent comparative loading analyses (Table 2; Figure 3G).

**TABLE 2:** Kinematic comparison of calculated SIJ ROM with Lindsey et al. (2014) and Zhang et al. (2022).

Motion	Present study (°)	Lindsey et al. (2014), mean ± SD (°)	Zhang et al. (2022) (°)
Left flexion-extension	2.56	1.91 ± 0.38	3.60
Left lateral bending	2.29	0.93 ± 0.25	2.30
Left axial rotation	2.30	1.58 ± 0.22	2.30
Right flexion-extension	2.86	2.75 ± 1.74	3.15
Right lateral bending	2.40	1.30 ± 1.11	1.65
Right axial rotation	2.59	1.79 ± 1.07	2.10

Lindsey et al. (2014) values were calculated from the individual intact-specimen data presented in Figure 3 (left SIJ, n = 4; right SIJ, n = 3).

**FIGURE 3:**
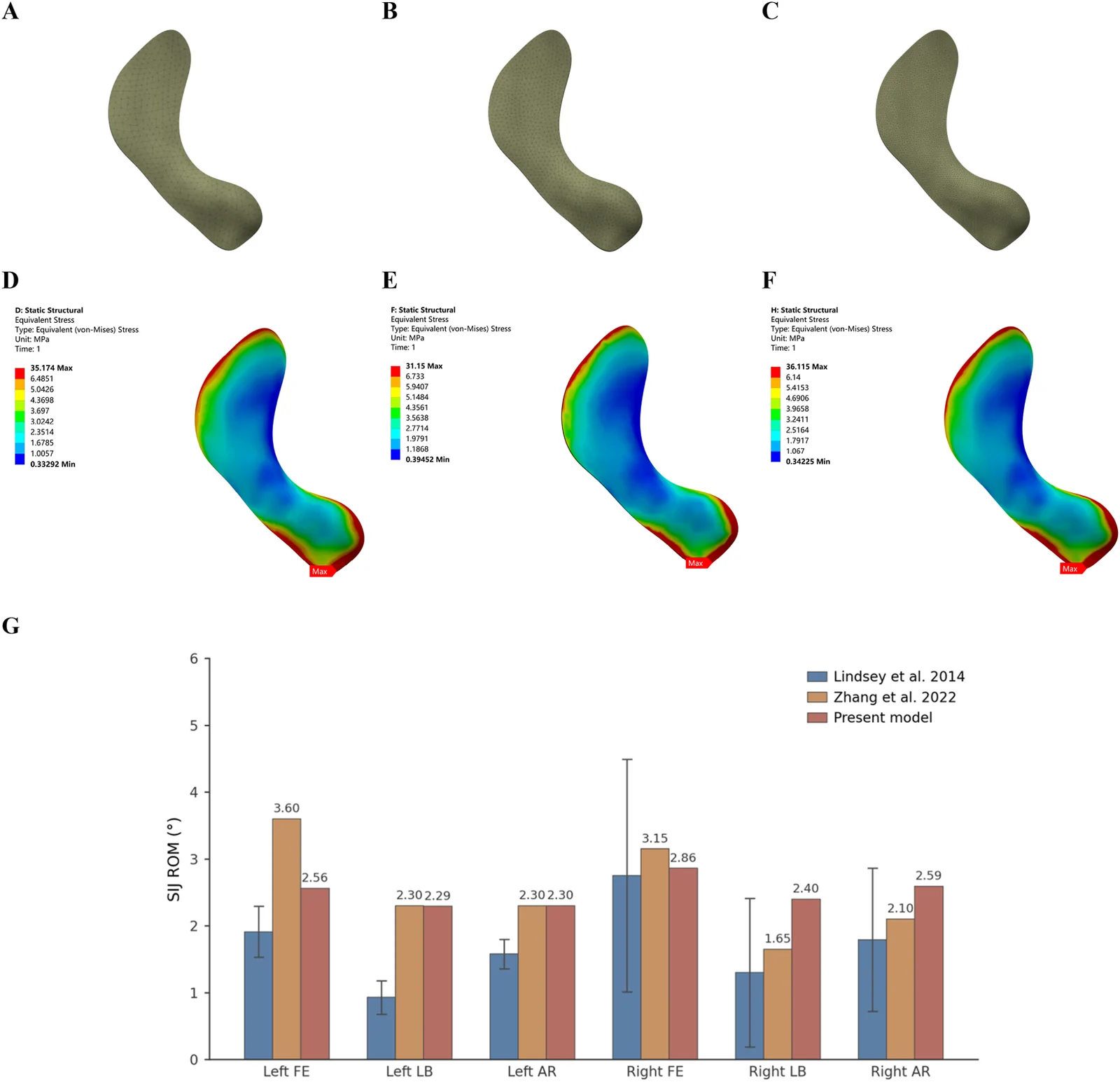
Kinematic comparison and local cartilage mesh assessment. **(A–C)** Local cartilage mesh sizes of 1.5, 1.0, and 0.5 mm; **(D–F)** corresponding cartilage VMS contours under OPM; **(G)** calculated SIJ ROM compared with published data.

Local cartilage mesh assessment showed peak VMS values of 31.150, 35.174, and 36.115 MPa for local cartilage mesh sizes of 1.5, 1.0, and 0.5 mm, respectively. Relative to the current 1.0-mm local cartilage mesh, the coarse and fine meshes differed by −11.44% and +2.68%, respectively. Further refinement from 1.0 to 0.5 mm changed the peak by only 2.68%, indicating reduced sensitivity of peak cartilage VMS to further local refinement (Table 3; Figures 3A–F). Probe checks placed the stress maxima in the same local anatomical region across the three local cartilage meshes.

**TABLE 3:** Three-level local cartilage mesh assessment under the OPM condition.

Mesh	Local cartilage mesh size (mm)	Nodes	Elements	Peak cartilage VMS (MPa)	Relative difference vs. current mesh (%)
Coarse	1.5	1,777,142	1,101,693	31.150	−11.44
Current	1.0	2,032,220	1,269,454	35.174	0.00
Fine	0.5	2,200,075	1,384,412	36.115	+2.68

Relative difference was calculated using the current 1.0-mm local cartilage mesh as the reference: (VMS_mesh - VMS_current)/VMS_current × 100%.

## Overall SIJ cartilage responses under different loading conditions

PCS and TMS produced nearly identical cartilage responses, with peak VMS values of 1.456 and 1.457 MPa, respectively, and a peak TD of 0.041 mm under both conditions. OPM produced the highest peak cartilage VMS and TD (35.174 MPa and 0.553 mm), followed by CDST-IM (11.057 MPa and 0.209 mm). The ROM of the left SIJ was 0.03°, 0.03°, 0.06°, and 0.03° under PCS, TMS, OPM, and CDST-IM, respectively, whereas the corresponding right SIJ ROM was 0.02°, 0.02°, 0.62°, and 0.21° (Table 4; Figures 4A–E). Principal-stress analysis identified both tensile and compressive stress components in the cartilage under all four conditions. The maximum principal stresses were 1.5476, 1.6921, 21.256, and 5.938 MPa, and the corresponding minimum principal stresses were −1.7196, −1.5554, −47.767, and −15.252 MPa, respectively. OPM therefore produced the largest positive and negative cartilage stress extrema (Table 4). Under OPM, the peak maximum principal stress was located at the lateral cartilage margin, where the local deformation pattern was consistent with lateral bulging under compression.

**TABLE 4:** Overall SIJ cartilage stress, TD, and SIJ ROM under different loading conditions.

Parameter	PCS	TMS	OPM	CDST-IM	Unit
Left SIJ ROM	0.03	0.03	0.06	0.03	°
Right SIJ ROM	0.02	0.02	0.62	0.21	°
Peak cartilage VMS	1.456	1.457	35.174	11.057	MPa
Cartilage maximum principal stress	1.5476	1.6921	21.256	5.938	MPa
Cartilage minimum principal stress	−1.7196	−1.5554	−47.767	−15.252	MPa
Peak cartilage TD	0.041	0.041	0.553	0.209	mm

**FIGURE 4:**
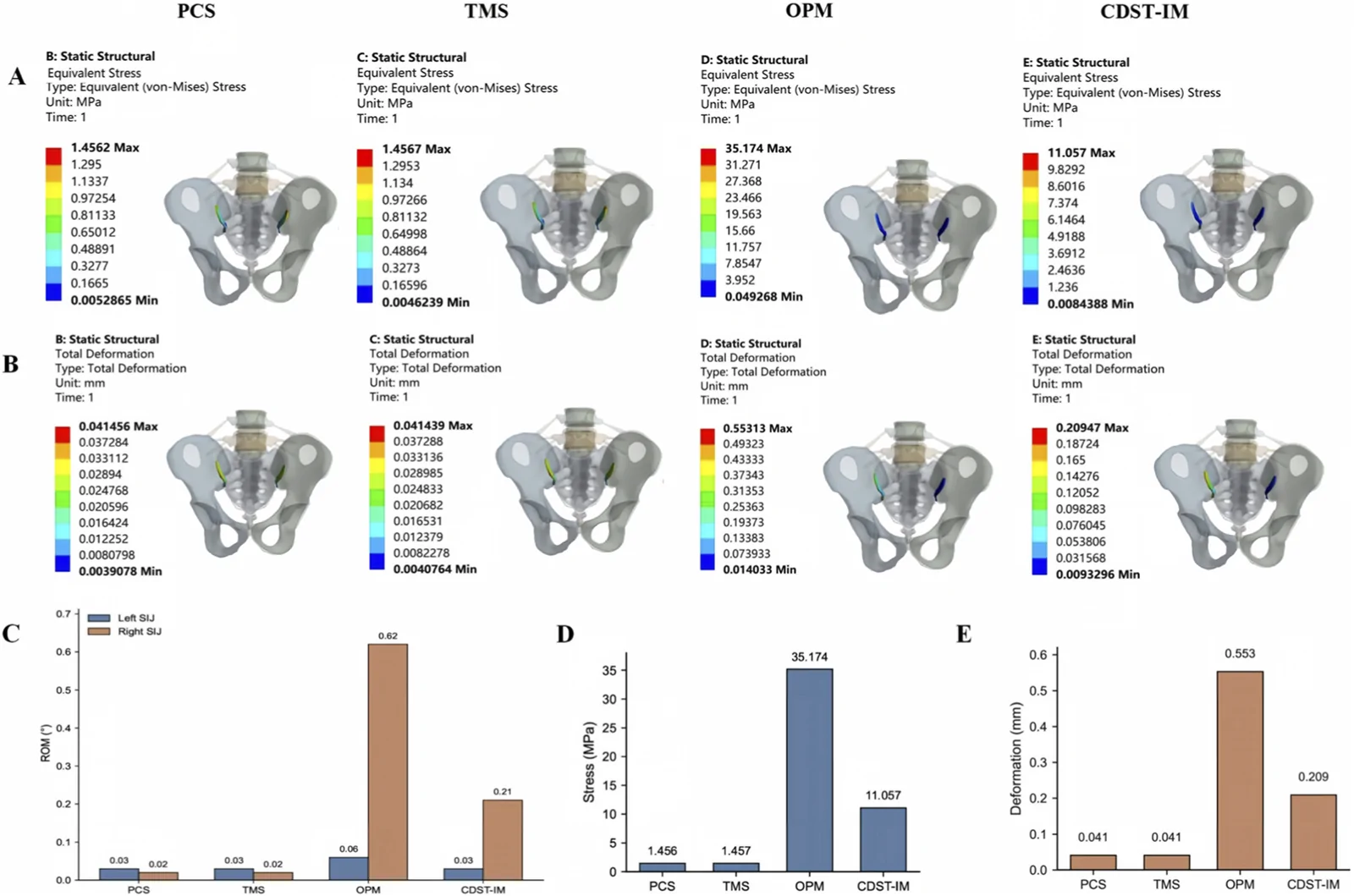
Overall SIJ cartilage and kinematic responses under the four loading conditions. **(A)** VMS and **(B)** TD contour maps of SIJ articular cartilage under PCS, TMS, OPM, and CDST-IM. Quantitative comparisons of **(C)** bilateral SIJ ROM, **(D)** peak cartilage VMS, and **(E)** peak cartilage TD.

### Surface-specific response of the sacral and iliac cartilage

The TD of the iliac-side and sacral-side cartilage surfaces was analyzed separately on both the left and right sides under the four loading conditions. On the left side, the sacral-side cartilage surface consistently showed greater TD than the iliac-side cartilage surface. Specifically, the TD values of the left iliac-side cartilage surface under PCS, TMS, OPM, and CDST-IM loading were 0.017, 0.018, 0.045, and 0.024 mm, respectively, whereas those of the left sacral-side cartilage surface were 0.037, 0.038, 0.065, and 0.036 mm, respectively.

On the right side, PCS and TMS showed the same relationship as that on the left side, with the sacral-side cartilage surface exhibiting greater TD than the iliac-side cartilage surface (0.041 vs. 0.022 mm under both loading conditions). In contrast, under OPM and CDST-IM loading, this relationship was reversed, and the iliac-side cartilage surface showed greater TD than the sacral-side cartilage surface. Under OPM loading, the TD of the right iliac-side and sacral-side cartilage surfaces were 0.553 and 0.265 mm, respectively, whereas under CDST-IM loading they were 0.209 and 0.146 mm, respectively (Table 5; Figures 5A–F).

**TABLE 5:** Surface-specific TD of iliac-side and sacral-side cartilage under different loading conditions.

Parameter	PCS	TMS	OPM	CDST-IM	Unit
Left iliac cartilage surface	0.017	0.018	0.045	0.024	mm
Left sacral cartilage surface	0.037	0.038	0.065	0.036	mm
Right iliac cartilage surface	0.022	0.022	0.553	0.209	mm
Right sacral cartilage surface	0.041	0.041	0.265	0.146	mm

**FIGURE 5:**
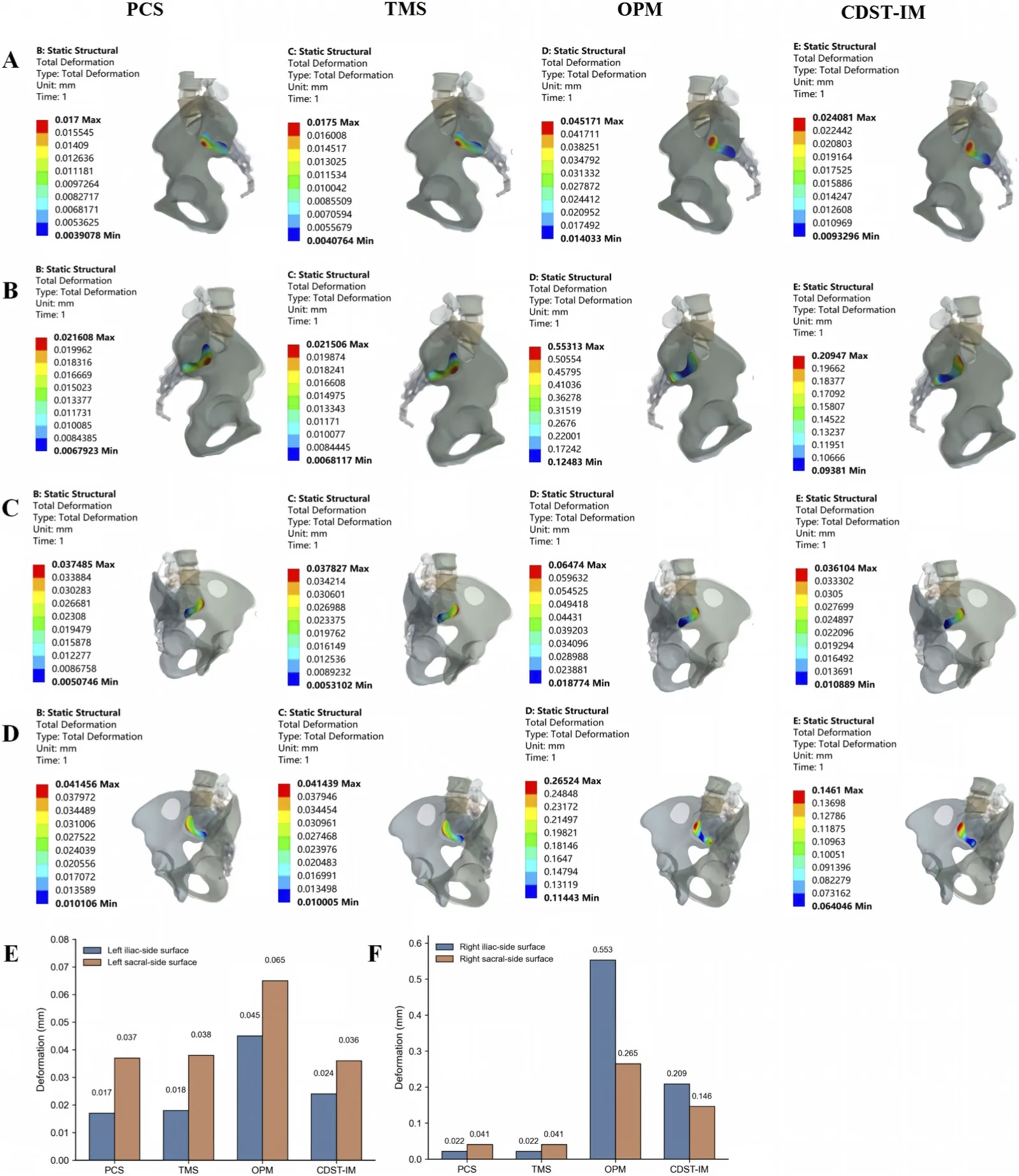
TD of the iliac-side and sacral-side SIJ cartilage surfaces under the four loading conditions. **(A–D)** TD contour maps of the left iliac-side, right iliac-side, left sacral-side, and right sacral-side cartilage surfaces, respectively, under PCS, TMS, OPM, and CDST-IM. Quantitative comparisons of iliac-side and sacral-side cartilage TD on the **(E)** left and **(F)** right sides.

Overall, OPM produced the most pronounced difference in TD between the iliac-side and sacral-side cartilage surfaces, particularly on the right side, where the iliac-side cartilage surface showed greater TD than the sacral-side surface. CDST-IM showed the same relationship but with a smaller TD difference.

### Iliolumbar ligament response under different loading conditions

Under the four types of loading conditions, the peak VMS values of the iliolumbar ligament were 4.106, 4.108, 4.717, and 1.306 MPa, respectively. The corresponding TD values were 1.529, 1.530, 1.604, and 0.451 mm, respectively. At the ligament level, PCS and TMS showed highly comparable response patterns, whereas OPM generated the greatest VMS and TD. In contrast, CDST-IM produced the lowest peak VMS and TD among the four loading conditions (Figures 6A-D). The maximum principal stress values of the iliolumbar ligament were 5.1224, 4.5064, 5.2906, and 1.6073 MPa under PCS, TMS, OPM, and CDST-IM, respectively (Table 6). Across the four conditions, the tensile peaks were accompanied by lower-magnitude localized compressive regions, reflecting spatially heterogeneous principal-stress distributions within the solid ligament bundles.

**FIGURE 6:**
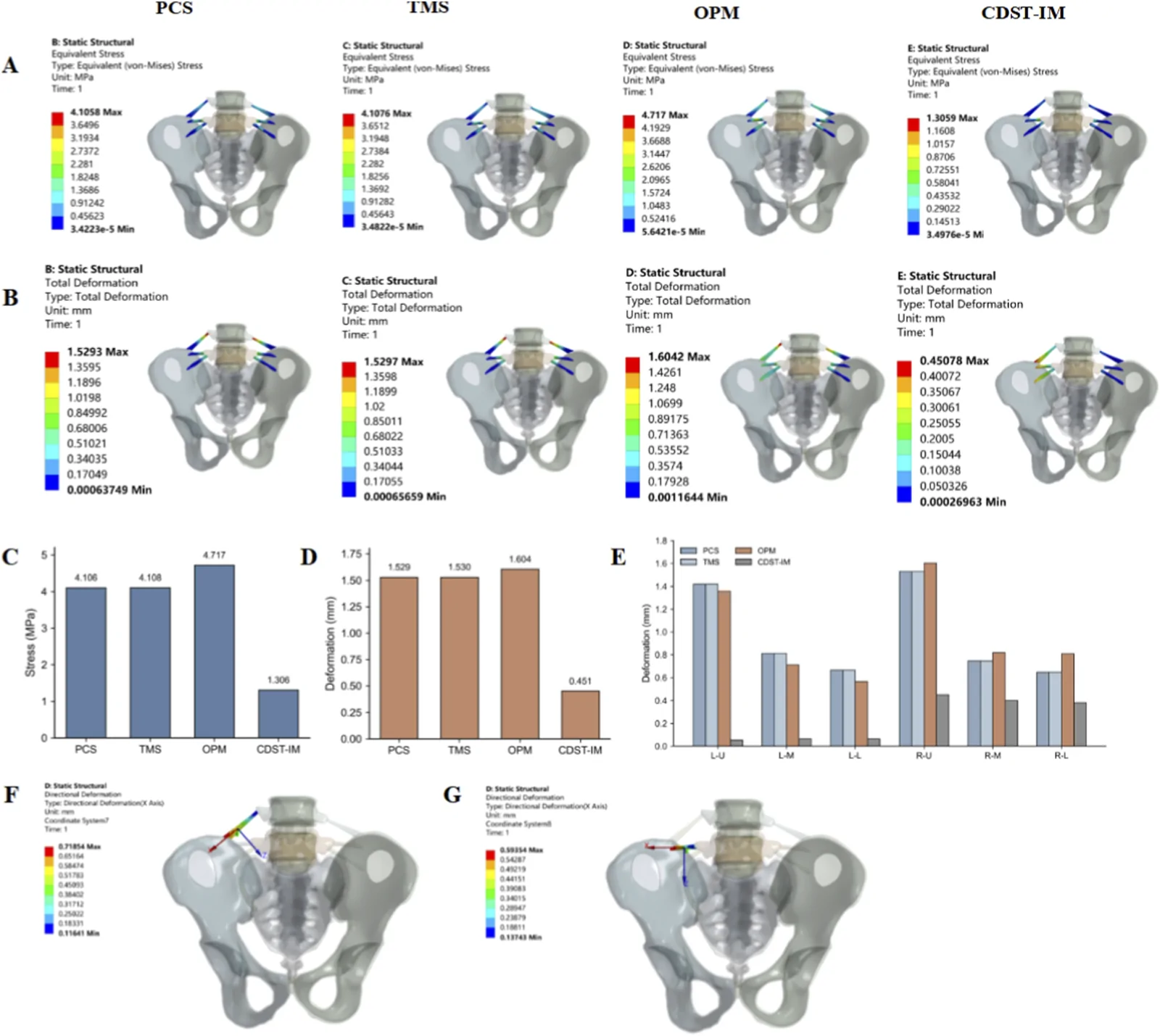
Iliolumbar ligament VMS, TD, and directional displacement. **(A)** VMS and **(B)** TD contour maps under PCS, TMS, OPM, and CDST-IM. Quantitative comparisons of **(C)** peak ligament VMS, **(D)** peak ligament TD, and **(E)** bundle-specific ligament TD. L-U, L-M, and L-L indicate the left upper, middle, and lower bundles, respectively; R-U, R-M, and R-L indicate the corresponding right-sided bundles; **(F,G)** Representative directional-displacement contour maps along the assigned local X-axis for the right upper **(F)** and right middle **(G)** bundles under OPM; the coordinate-system triads indicate the corresponding local-axis orientations.

**TABLE 6:** VMS, maximum principal stress, TD, and maximum directional displacement of the iliolumbar ligament under different loading conditions.

Parameter	PCS	TMS	OPM	CDST-IM	Unit
Peak ligament VMS	4.106	4.108	4.717	1.306	MPa
Ligament maximum principal stress	5.1224	4.5064	5.2906	1.6073	MPa
Peak ligament TD	1.529	1.530	1.604	0.451	mm
Left upper bundle TD	1.421	1.421	1.358	0.054	mm
Left middle bundle TD	0.811	0.811	0.711	0.064	mm
Left lower bundle TD	0.665	0.665	0.564	0.063	mm
Right upper bundle TD	1.529	1.530	1.604	0.451	mm
Right middle bundle TD	0.746	0.747	0.819	0.400	mm
Right lower bundle TD	0.648	0.648	0.810	0.382	mm
Left upper bundle directional displacement	0.31529	0.31542	0.53616	0.041772	mm
Left middle bundle directional displacement	0.18772	0.18784	0.26618	0.048501	mm
Left lower bundle directional displacement	0.16159	0.16176	0.27948	0.037681	mm
Right upper bundle directional displacement	0.27214	0.27223	0.71854	0.13596	mm
Right middle bundle directional displacement	0.15634	0.15630	0.59354	0.18737	mm
Right lower bundle directional displacement	0.16521	0.16523	0.58541	0.12418	mm

The TD values of the individual iliolumbar ligament bundles are summarized in Table 6 and Figure 6E. Across the four loading conditions, a predominantly upper-bundle pattern was observed, particularly on the right side and under OPM. This pattern was most evident under OPM loading, especially in the right upper bundle. In contrast, CDST-IM was associated with lower TD in all bundles, although the right-sided bundles showed greater TD than the corresponding left-sided bundles. Maximum directional displacement along the assigned local X-axis was greatest under OPM for all six bundles (Table 6). The right upper, right middle, and right lower bundles reached 0.71854, 0.59354, and 0.58541 mm, respectively; the assigned local coordinate systems for the right upper and right middle bundles are shown in Figures 6F,G. These direction-specific results indicate that OPM produced the most pronounced ligament motion along the principal loading direction, particularly in the right-sided bundles.

Relative to PCS, TMS changed peak ligament VMS and TD by only 0.04% and 0.03%, respectively. OPM increased peak ligament VMS and TD by 14.89% and 4.90%, respectively; among the individual bundles, the right lower bundle showed the greatest TD increase (+25.08%). CDST-IM reduced peak ligament VMS and TD by 68.19% and 70.52%, respectively, and reduced TD in all six bundles (Table 7). Taken together with the principal-stress and directional-displacement findings, these results indicate that OPM produced the greatest overall iliolumbar ligament response, whereas CDST-IM produced a comparatively smaller ligament response.

**TABLE 7:** Relative changes in iliolumbar ligament VMS and TD compared with PCS.

Parameter	TMS vs. PCS	OPM vs. PCS	CDST-IM vs. PCS
Peak ligament VMS	+0.04%	+14.89%	−68.19%
Peak ligament TD	+0.03%	+4.90%	−70.52%
Left upper bundle TD	+0.01%	−4.43%	−96.22%
Left middle bundle TD	+0.02%	−12.39%	−92.09%
Left lower bundle TD	+0.02%	−15.21%	−90.59%
Right upper bundle TD	+0.03%	+4.90%	−70.52%
Right middle bundle TD	+0.03%	+9.77%	−46.35%
Right lower bundle TD	+0.04%	+25.08%	−41.00%

PCS, was used as the reference. Relative change = (condition − PCS)/PCS × 100%.

### Overall comparison of loading patterns

Among the conditions, PCS and TMS produced nearly identical magnitudes in the reported outputs in the SIJ-iliolumbar ligament complex. In contrast, OPM and CDST-IM both increased the mechanical response of the SIJ relative to PCS and TMS, but with different magnitudes. OPM produced the highest cartilage VMS, TD, and right SIJ ROM, whereas CDST-IM showed intermediate values that remained lower than those observed under OPM.

Under both OPM and CDST-IM, the iliac-side cartilage surface showed greater TD than the sacral-side cartilage surface on the right side, whereas PCS and TMS showed the opposite relationship. In the iliolumbar ligament, OPM generated the greatest VMS and TD, while CDST-IM produced the lowest ligament response among the four loading conditions. Taken together, these findings indicate that OPM and CDST-IM induced distinct biomechanical response patterns, despite both modifying the loading environment of the SIJ.

## Discussion

The results of this study showed that the four loading conditions produced different mechanical responses in the SIJ-iliolumbar ligament complex. PCS and TMS produced nearly identical responses among the evaluated parameters, suggesting that under bilateral iliac constraint, simply reversing a 300 N axial load produced only small changes. In contrast, OPM produced the strongest overall biomechanical response, including the highest cartilage VMS, cartilage TD, right SIJ ROM, and iliolumbar ligament VMS and TD. CDST-IM also altered the mechanical environment of the SIJ, but its response pattern was different from OPM: the articular cartilage responses increased relative to PCS/TMS, while the ligament responses were relatively low. These results indicate that the mechanical effect of manipulative loading depends on the combined effects of loading direction, pelvic constraint, and local force application rather than on axial load direction alone.

These results should be understood in conjunction with the biomechanical characteristics of SIJ itself. Unlike a freely mobile synovial joint, the SIJ mainly acts as a constrained load transfer joint (Vleeming et al., 1990a; Vleeming et al., 1990b). *In vivo*, its stability mainly depends on joint congruence, articular surface morphology, frictional resistance and strong ligamentous constraints. Snijders et al. (1993) described this stability mechanism as a self-bracing mechanism, that is, under load, SIJ is more inclined to obtain mechanical strengthening rather than free displacement. Subsequently, this concept was further developed into the framework of form closure and force closure, emphasizing the combined effects of joint geometry and compressive support around the joint in maintaining SIJ stability (Vleeming et al., 2012; Vleeming and Schuenke, 2019). *In vivo* evidence broadly supports this low-motion biomechanical profile. Although Smidt et al. (1995) reported measurable angular SIJ motion in reciprocal straddle positions, those findings were obtained under highly asymmetric end-range postures rather than routine physiological loading. By contrast, Sturesson et al. (2000) showed that SIJ motion during the standing hip flexion test was very small, and Walker (1992) likewise noted that SIJ motion is inherently limited and difficult to quantify precisely because of the joint’s anatomical configuration. More directly, Tullberg et al. (1998) reported no measurable positional change in the SIJ after manipulation. In the present study, both OPM and CDST-IM substantially increased right SIJ ROM relative to baseline conditions, but the absolute magnitude remained within a sub-degree range (0.62° under OPM and 0.21° under CDST-IM). Although OPM and CDST-IM represent manipulative loading conditions, their ROM values were compatible with the small angular magnitudes reported for intact SIJs under physiological and experimental loading (Hammer et al., 2019; Xu et al., 2023b). Even sub-degree motion may affect joint constraint and ligament response, with potential implications for lumbopelvic load transmission (Pool-Goudzwaard et al., 2003; Ivanov et al., 2009).

However, limited SIJ motion does not imply limited mechanical relevance. In the SIJ, the biomechanical significance of motion may depend not only on displacement amplitude, but also on potential changes in joint constraint and load-transfer patterns. In this context, ligament integrity and boundary conditions are critical determinants of SIJ mechanical behavior (Lee et al., 2017; Shih et al., 2018; Cross et al., 2018). Pool-Goudzwaard et al. (2003) demonstrated that sequential transection of the iliolumbar ligament significantly increased sagittal SIJ mobility, indicating that ligament restraint contributes directly to joint constraint. Leong et al. (1987) likewise showed that the iliolumbar ligament plays an important role in maintaining lumbosacral stability, while Dujardin et al. (2002) reported that even small SIJ micromotion may reflect meaningful posterior pelvic instability. Vrahas et al. (1995) further emphasized that pelvic stability depends strongly on ligamentous support rather than on osseous architecture alone. In the present study, this stabilizing role was reflected in the distinct ligament responses under the two manipulative loading conditions. OPM produced the highest ligament VMS and TD, the highest maximum-principal-stress peak, and the greatest directional displacement along the assigned local X-axis in all six bundles. The maximum-principal-stress fields showed positive tensile peaks with lower-magnitude localized compressive regions, while the directional-displacement results demonstrated pronounced bundle-specific motion, particularly in the right-sided bundles under OPM. CDST-IM produced substantially smaller ligament stress and displacement responses. Together, these findings show that ligament behavior varied with the loading configuration and complemented the ROM results in characterizing the mechanical response of the SIJ-iliolumbar ligament complex.

From this perspective, the findings for the sacral and iliac cartilage surfaces of the present study are particularly informative. Because SIJ cartilage has distinct structural and biomechanical characteristics, displacement of the sacral and iliac cartilage surfaces may provide useful information for characterizing SIJ surface motion (Nordberg et al., 2024). Under PCS and TMS, TD on the right side was greater on the sacral cartilage surface than on the iliac cartilage surface. In the OPM and CDST-IM conditions, greater cartilage displacement was observed on the iliac side. This indicates a change in which cartilage surface showed greater displacement, rather than a simple increase in overall joint displacement. This interpretation is consistent with the finite element study by Xu et al. (2020), who reported small SIJ displacements but distinct ligament strain and stress responses under different manipulation modes. The present model further indicates that OPM and CDST-IM produced a similar shift in the cartilage surface showing greater displacement, although the magnitude of this shift and the difference between sides varied between the two conditions. In both manipulative conditions, the surface showing greater TD changed from the sacral side to the iliac side. However, this shift was much more pronounced under OPM, where right iliac-side TD was more than twice the right sacral-side TD. By comparison, the difference between iliac-side and sacral-side TD was smaller under CDST-IM. Beyond these surface-displacement patterns, maximum and minimum principal stress analyses clarified the associated cartilage stress state. Positive maximum principal stresses and negative minimum principal stresses were observed under all four conditions, indicating the presence of both tensile and compressive stress components. OPM produced the highest maximum principal stress (21.256 MPa), followed by CDST-IM (5.938 MPa), while the minimum principal stress results further demonstrated a compressive component within the cartilage. These principal-stress findings complement the surface-specific TD results by characterizing both cartilage surface motion and local stress states.

The iliolumbar ligament data provide further support for the view that OPM and CDST-IM represent mechanically distinct maneuvers rather than a single mechanism operating at different intensities. OPM generated the highest iliolumbar ligament VMS and TD, and the response was most evident in the right upper bundle. An upper-bundle dominant pattern was observed overall; however, under OPM the greatest absolute TD occurred in the right upper bundle, whereas the left upper bundle remained relatively large in absolute terms without further increase relative to PCS/TMS. By contrast, CDST-IM produced the lowest ligament response among the four loading conditions, even though SIJ cartilage VMS, TD, and right SIJ ROM were all clearly increased relative to PCS and TMS. This divergence is mechanically meaningful. OPM combines upward loading at L4 with posterior rotation of the right ilium, thereby creating a coupled rotation-traction pattern that appears to be associated with a greater iliolumbar ligament response. CDST-IM, in contrast, applies a posterior force along the SIJ direction and appears to modify the articular response with relatively limited ligament response. Accordingly, OPM may be interpreted as producing concurrent SIJ and periarticular ligament responses, whereas CDST-IM showed a distinct articular-ligamentous response pattern with comparatively smaller iliolumbar ligament responses. Importantly, a higher internal stress state in this model should be interpreted as increased local load concentration rather than as evidence of large-amplitude joint relocation.

Taken together, these findings describe configuration-specific mechanical responses and cannot determine whether clinical SIJ manipulation involves joint repositioning. Within a joint system that is inherently stabilized by form closure, force closure, and strong periarticular restraint, the modeled changes reflect limited micromotion within the prescribed constraints. Tullberg et al. (1998) found no measurable positional change in the SIJ after manipulation, supporting the view that pain relief does not require radiographically demonstrable joint resetting. Ivanov et al. (2009) showed that altered lumbosacral load transfer after lumbar fusion can increase both SIJ motion and SIJ stress, indicating that modification of the surrounding mechanical environment alone may be sufficient to alter SIJ behavior. Szadek et al. (2010) identified nociceptive structures in SIJ cartilage and adjacent ligamentous tissues, suggesting that local stress redistribution may be clinically relevant even when observable joint motion remains small. This interpretation is compatible with, but does not overstate, the available treatment literature. Trager et al. (2024) concluded in a recent systematic review and meta-analysis that manual therapy may improve disability in SIJ pain syndrome, while also emphasizing heterogeneity in the current evidence base. Nejati et al. (2019) reported that both manipulation and exercise therapy improved outcomes in patients with SIJ dysfunction, and Javadov et al. (2021) likewise found that manual therapy combined with sacroiliac and lumbar exercises improved pain and function in selected patients. Notably, none of these studies demonstrated that clinical success required radiographically verifiable SIJ repositioning. These clinical observations are compatible with the limited SIJ motion and altered periarticular mechanical responses observed in the present model; however, whether these modeled responses contribute to clinical benefit cannot be determined from this single-subject, static analysis.

Several limitations should be noted. First, the model was based on one healthy adult male and cannot represent age-, sex-, or pathology-related variability. The clinical maneuvers were represented by simplified terminal loading conditions rather than their full dynamic processes. Because each condition used technique-specific loading and constraint definitions, between-condition differences should be interpreted as configuration-specific mechanical responses, and direct manipulation-specific experimental validation of the cartilage and ligament outputs could not be performed in the absence of directly matched experimental data. Second, a three-level local cartilage mesh assessment under OPM showed a 2.68% difference in peak VMS between the current and fine meshes; however, ROM/TD convergence and contact-formulation sensitivity were not assessed, and absolute peak VMS values should therefore still be interpreted cautiously. Third, the static model omitted active muscle forces and modeled SIJ cartilage as linear elastic without depth-dependent, anisotropic, biphasic, or time-dependent behavior. Cartilage-modulus sensitivity was not assessed, so the effects of lower moduli on absolute stresses and comparisons remain untested. The iliolumbar ligaments were simplified as linear isotropic solids without pretension or time-dependent behavior; ligament pretension may also influence SIJ model outputs (Heyland et al., 2025). The frictionless, no-separation contact definition and rigid sacral fixation represent additional modeling assumptions associated with the prescribed terminal loading states. Future analyses incorporating alternative contact formulations and compliant support conditions may further clarify whether the observed tensile cartilage stresses are influenced by the adopted contact assumptions. The principal-stress and directional-displacement analyses provided complementary characterization of local stress states and bundle-level motion along the assigned ligament directions. Future evaluation of attachment-to-attachment elongation, strain, and insertion force may further quantify ligament tensile deformation and load transfer. Finally, the subject-specific geometry was reconstructed from CT images with a 5-mm slice thickness, which limited through-plane anatomical resolution. Future studies using higher-resolution imaging or additional validation data may further improve the anatomical representation of subject-specific SIJ structures.

## Conclusion

In summary, our present finite element analysis indicates that OPM and CDST-IM both alter the biomechanical environment of the SIJ through distinct response patterns. Within the present model, OPM showed a more pronounced concurrent articular-ligamentous response, whereas CDST-IM showed increased articular responses with comparatively smaller iliolumbar ligament responses. On the right side, the cartilage surface showing greater TD changed from the sacral surface under PCS/TMS to the iliac surface under OPM/CDST-IM. Overall, the modeled responses were consistent with limited SIJ micromotion under the prescribed loading conditions, while their relevance to clinical repositioning mechanisms remains beyond the scope of the present simulations.

## Data Availability

The data supporting the findings of this study are available from the corresponding author upon reasonable request. Raw CT imaging data are not publicly available due to participant privacy and ethical considerations.

## References

[B1] Bruna-Rosso C. Arnoux P. J. Bianco R. J. Godio-Raboutet Y. Fradet L. Aubin C. E. (2016). Finite element analysis of sacroiliac joint fixation under compression loads. Int. J. Spine Surg. 10, 16. 10.14444/3016 27441174 PMC4943166

[B2] Buchanan P. Vodapally S. Lee D. W. Hagedorn J. M. Bovinet C. Strand N. (2021). Successful diagnosis of sacroiliac joint dysfunction. J. Pain Res. 14, 3135–3143. 10.2147/JPR.S327351 34675642 PMC8517984

[B3] Cross W. W. Berven S. H. Slater N. Lehrman J. N. Newcomb A. G. U. S. Kelly B. P. (2018). *In vitro* biomechanical evaluation of a novel, minimally invasive, sacroiliac joint fixation device. Int. J. Spine Surg. 12, 587–594. 10.14444/5072 30364882 PMC6198630

[B4] Dujardin F. H. Roussignol X. Hossenbaccus M. Thomine J. M. (2002). Experimental study of the sacroiliac joint micromotion in pelvic disruption. J. Orthop. Trauma 16, 99–103. 10.1097/00005131-200202000-00005 11818804

[B5] Eichenseer P. H. Sybert D. R. Cotton J. R. (2011). A finite element analysis of sacroiliac joint ligaments in response to different loading conditions. Spine 36, E1446–E1452. 10.1097/BRS.0b013e31820bc705 21311405

[B6] Guo L. X. Li W. J. (2020). Finite element modeling and static/dynamic validation of the thoracolumbar-pelvic segment. Comput. Methods Biomech. Biomed. Engin. 23, 69–80. 10.1080/10255842.2019.1699543 31813282

[B7] Hammer N. Scholze M. Kibsgård T. Klima S. Schleifenbaum S. Seidel T. (2019). Physiological *in vitro* sacroiliac joint motion: a study on three-dimensional posterior pelvic ring kinematics. J. Anat. 234, 346–358. 10.1111/joa.12924 30536830 PMC6365483

[B8] He W. Li J. L. Wang J. Y. He D. Song K. (2024). Optimization of traction parameters for lumbar scoliosis. BMC Musculoskelet. Disord. 25, 472. 10.1186/s12891-024-07583-y 38880874 PMC11181601

[B9] Heyland M. Schmidt H. Schomig F. Maikath D. Deppe D. Pumberger M. (2025). Ligament pre-tension determines outcome in sacroiliac joint in silicon modelling. Med. Biol. Eng. Comput. 63, 3321–3333. 10.1007/s11517-025-03396-w 40526189 PMC12634734

[B10] Hu P. Wu T. Wang H. Z. Qi X. Z. Yao J. Cheng X. D. (2017). Influence of different boundary conditions in finite element analysis on pelvic biomechanical load transmission. Orthop. Surg. 9, 115–122. 10.1111/os.12315 28300359 PMC6584104

[B11] Ivanov A. A. Kiapour A. Ebraheim N. A. Goel V. (2009). Lumbar fusion leads to increases in angular motion and stress across sacroiliac joint: a finite element study. Spine 34, E162–E169. 10.1097/BRS.0b013e3181978ea3 19247155

[B12] Javadov A. Ketenci A. Aksoy C. (2021). The efficiency of manual therapy and sacroiliac and lumbar exercises in patients with sacroiliac joint dysfunction syndrome. Pain Physician 24, 223–233. 10.36076/ppj.2021/24/223 33988941

[B13] Kiapour A. Joukar A. Elgafy H. Erbulut D. U. Agarwal A. K. Goel V. K. (2020). Biomechanics of the sacroiliac joint: anatomy, function, biomechanics, sexual dimorphism, and causes of pain. Int. J. Spine Surg. 14, S3–S13. 10.14444/6077 32123652 PMC7041664

[B14] Lee C. H. Hsu C. C. Huang P. Y. (2017). Biomechanical study of different fixation techniques for the treatment of sacroiliac joint injuries using finite element analyses and biomechanical tests. Comput. Biol. Med. 87, 250–257. 10.1016/j.compbiomed.2017.06.007 28618337

[B15] Leong J. C. Luk K. D. Chow D. H. Woo C. W. (1987). The biomechanical functions of the iliolumbar ligament in maintaining stability of the lumbosacral junction. Spine 12, 669–674. 10.1097/00007632-198709000-00005 3686218

[B16] Li J. Li Y. Ping R. Zhang Q. Chen H. Y. Lin D. (2022). Biomechanical analysis of sacroiliac joint motion following oblique-pulling manipulation with or without pubic symphysis injury. Front. Bioeng. Biotechnol. 10, 960090. 10.3389/fbioe.2022.960090 36204470 PMC9530983

[B17] Lindsey D. P. Perez-Orribo L. Rodriguez-Martinez N. Reyes P. M. Newcomb A. Cable A. (2014). Evaluation of a minimally invasive procedure for sacroiliac joint fusion-an *in vitro* biomechanical analysis of initial and cycled properties. Med. Devices 7, 131–137. 10.2147/MDER.S63499 24868175 PMC4031207

[B18] Lindsey D. P. Kiapour A. Yerby S. A. Goel V. K. (2015). Sacroiliac joint fusion minimally affects adjacent lumbar segment motion: a finite element study. Int. J. Spine Surg. 9, 64. 10.14444/2064 26767156 PMC4710165

[B19] Long C. H. Fan D. H. (2019). Long shi zhi ji liao fa [Long's spinal therapy]. 2nd ed. Guangzhou: Guangdong Science and Technology Press, 141–145.

[B20] Long C. H. Zhong S. Y. Wang T. C. (2004). Pelvic rotatory displacement syndrome. J. Cervicodynia Lumbodynia 3, 198–202.

[B21] Nejati P. Safarcherati A. Karimi F. (2019). Effectiveness of exercise therapy and manipulation on sacroiliac joint dysfunction: a randomized controlled trial. Pain Physician 22, 53–61. 10.36076/ppj/2019.22.53 30700068

[B22] Newman D. P. Soto A. T. (2022). Sacroiliac joint dysfunction: diagnosis and treatment. Am. Fam. Physician 105, 239–245. 35289578

[B23] Nordberg R. C. Hight J. M. Kim A. N. Meka R. S. Elder B. D. Hu J. C. (2024). Biomechanical, biochemical, and histological characterization of sacroiliac joint cartilage in the Yucatan minipig. J. Mech. Behav. Biomed. Mater. 157, 106658. 10.1016/j.jmbbm.2024.106658 39018919 PMC11516651

[B24] Pool-Goudzwaard A. L. van Dijke G. H. Mulder P. G. H. Spoor C. W. Snijders C. J. Stoeckart R. (2003). The iliolumbar ligament: its influence on stability of the sacroiliac joint. Clin. Biomech. 18, 99–105. 10.1016/S0268-0033(02)00179-1 12550807

[B25] Shahraki N. M. Fatemi A. Goel V. K. (2015). On the use of biaxial properties in modeling annulus as a holzapfel-gasser-ogden material. Front. Bioeng. Biotechnol. 3, 69. 10.3389/fbioe.2015.00069 PMC4453479 26090359

[B26] Shi S. Xu S. Zhou X. Wang Y. Han C. Chen M. (2026). Effect of sacrum-stabilized iliac traction on the stress and displacement of sacroiliac joint and iliolumbar ligament. J. Biomed. Eng. 43 (1), 154–160. 10.7507/1001-5515.202509007 PMC12948545 41760215

[B27] Shih Y. C. Beaubien B. P. Chen Q. Sembrano J. N. (2018). Biomechanical evaluation of sacroiliac joint fixation with decortication. Spine J. 18, 1241–1249. 10.1016/j.spinee.2018.02.016 29481980

[B28] Smidt G. L. McQuade K. Wei S. H. Barakatt E. (1995). Sacroiliac kinematics for reciprocal straddle positions. Spine 20, 1047–1054. 10.1097/00007632-199505000-00011 7631234

[B29] Snijders C. J. Vleeming A. Stoeckart R. (1993). Transfer of lumbosacral load to iliac bones and legs. Part 1: biomechanics of self-bracing of the sacroiliac joints and its significance for treatment and exercise. Clin. Biomech. 8, 285–294. 10.1016/0268-0033(93)90002-Y 23916048

[B30] Sturesson B. Uden A. Vleeming A. (2000). A radiostereometric analysis of movements of the sacroiliac joints during the standing hip flexion test. Spine 25, 364–368. 10.1097/00007632-200002010-00018 10703111

[B31] Szadek K. M. Hoogland P. V. J. M. Zuurmond W. W. A. de Lange J. J. Perez R. S. G. M. (2010). Possible nociceptive structures in the sacroiliac joint cartilage: an immunohistochemical study. Clin. Anat. 23, 192–198. 10.1002/ca.20908 20014392

[B32] Trager R. J. Baumann A. N. Rogers H. Tidd J. Orellana K. Preston G. (2024). Efficacy of manual therapy for sacroiliac joint pain syndrome: a systematic review and meta-analysis of randomized controlled trials. J. Man. Manip. Ther. 32, 561–572. 10.1080/10669817.2024.2316420 38353102 PMC11578406

[B33] Tullberg T. Blomberg S. Branth B. Johnsson R. (1998). Manipulation does not alter the position of the sacroiliac joint: a roentgen stereophotogrammetric analysis. Spine 23, 1124–1128. 10.1097/00007632-199805150-00010 9615363

[B34] Vleeming A. Schuenke M. (2019). Form and force closure of the sacroiliac joints. PM R. 11, S24–S31. 10.1002/pmrj.12205 31218826

[B35] Vleeming A. Stoeckart R. Volkers A. C. Snijders C. J. (1990a). Relation between form and function in the sacroiliac joint. Part I: clinical anatomical aspects. Spine 15, 130–132. 10.1097/00007632-199002000-00016 2326706

[B36] Vleeming A. Volkers A. C. Snijders C. J. Stoeckart R. (1990b). Relation between form and function in the sacroiliac joint. Part II: biomechanical aspects. Spine 15, 133–136. 10.1097/00007632-199002000-00017 2326707

[B37] Vleeming A. Schuenke M. D. Masi A. T. Carreiro J. E. Danneels L. Willard F. H. (2012). The sacroiliac joint: an overview of its anatomy, function and potential clinical implications. J. Anat. 221, 537–567. 10.1111/j.1469-7580.2012.01564.x 22994881 PMC3512279

[B38] Vrahas M. Hern T. C. Diangelo D. Kellam J. Tile M. (1995). Ligamentous contributions to pelvic stability. Orthopedics 18, 271–274. 10.3928/0147-7447-19950301-09 7761317

[B39] Walker J. M. (1992). The sacroiliac joint: a critical review. Phys. Ther. 72, 903–916. 10.1093/ptj/72.12.903 1454866

[B40] Xu Z. Li Y. Zhang S. Liao L. Wu K. Feng Z. (2020). A finite element analysis of sacroiliac joint displacements and ligament strains in response to three manipulations. BMC Musculoskelet. Disord. 21, 709. 10.1186/s12891-020-03735-y 33115467 PMC7594473

[B41] Xu Z. Feng Z. Zhang Z. Zhang K. Li Y. (2023a). Manipulations of oblique pulling affect sacroiliac joint displacements and ligament strains: a finite element analysis. J. Healthc. Eng. 2023, 2840421. 10.1155/2023/2840421 36636670 PMC9831713

[B42] Xu Z. Huang Z. Zhang Z. Feng Z. Yan Y. Zhu Q. (2023b). Effects of manipulations of oblique pulling on the biomechanics of the sacroiliac joint: a cadaveric study. BMC Musculoskelet. Disord. 24, 55. 10.1186/s12891-023-06175-6 36683034 PMC9869564

[B43] Zhang S. Q. Li N. Q. Qi J. Li Y. K. (2018). Historical evolution of sacroiliac joint malposition based on bibliometric analysis. Chin. J. Tissue Eng. Res. 22, 4316–4321. 10.3969/j.issn.2095-4344.0345

[B44] Zhang S. Chen Y. Ren R. Jiang S. Cao Y. Li Y. (2022). Quantitative study on the biomechanical mechanism of sacroiliac joint subluxation: a finite element study. J. Orthop. Res. 40, 1223–1235. 10.1002/jor.25132 34185334

[B45] Zhou X. Wang Y. Lin J. Chen H. Ren Q. Shi S. (2025). Effects of oblique-pulling manipulation on stress and displacement of sacroiliac joints and iliolumbar ligaments. J. Med. Biomechanics 40 (6), 1638–1645. 10.16156/j.1004-7220.2025.06.034

[B46] Zhu B. Fan B. H. Wang P. Ying X. M. Xu Q. Z. Guo S. Q. (2012). Clinical observation on curative effect of Wa-shi pulling on treating sacroiliac joint pain. Chin. Archives Traditional Chin. Med. 30, 1607–1609.

